# A Human‐Centred Co‐Design Framework for Developing a Web‐Based Platform to Engage With Rural Australian Communities: Addressing the Complex Issue of Healthy Food Access

**DOI:** 10.1111/ajr.70028

**Published:** 2025-03-20

**Authors:** Cindy Needham, Jane Jacobs, Christina Zorbas, Laura Alston, Tracy Schumacher, Penny Fraser, Ana Horta, Michael Johnstone, Douglas Creighton, Alison Koschel, Leanne J. Brown, Annabelle Williams, Judy Coates, Gina Vereker, Carmen Vargas, Claudia Strugnell, Tari Forrester‐Bowling, Kristy A Bolton, Steven Allender

**Affiliations:** ^1^ Global Centre for Preventive Health and Nutrition, Institute for Health Transformation Deakin University Geelong Victoria Australia; ^2^ Research Unit Colac Area Health Colac Victoria Australia; ^3^ Deakin Rural Health, Faculty of Health Deakin University Warrnambool Victoria Australia; ^4^ Department of Rural Health University of Newcastle Tamworth New South Wales Australia; ^5^ Gulbali Institute Charles Sturt University Albury New South Wales Australia; ^6^ Institute for Intelligent Systems Research and Innovation Deakin University (IISRI) Waurn Ponds Victoria Australia; ^7^ Hunter New England and Central Coast Primary Health Network Tamworth New South Wales Australia; ^8^ Hunter Medical Research Institute Food and Nutrition Research Program New Lambton Heights New South Wales Australia; ^9^ Sora Community Services Tamworth New South Wales Australia; ^10^ Tamworth Regional Council Tamworth New South Wales Australia; ^11^ Institute for Physical Activity and Nutrition Deakin University Waurn Ponds Victoria Australia

**Keywords:** food environment, food retail, health inequalities, human‐centred design, public health nutrition, rural

## Abstract

**Objective:**

This report presents the co‐design framework for developing, prototyping, testing and implementing a Web‐based Platform (WBP) that will use participatory approaches to engage rural communities.The WBP will be used to understand the unique factors influencing access to healthy and unhealthy foods and to generate potential solutions for promoting healthier diets.

**Methods:**

A human‐centred design (HSD) approach will be used to ideate, prototype, test and implement the WBP.

**Design:**

Participatory Action Research.

**Setting:**

Two rural local government areas in Australia.

**Participants:**

Participants will include key stakeholders from each local government's relevant public health organisations, in addition to community members.

**Main Outcome Measures:**

Reach (number, cohort representation and geographic spread) of active participants in the co‐design process, community members that used the WBP tool and adoption (completion of WBP activities).

**Results:**

A usable platform for communities to generate local solutions to drive change for diverse populations within rural communities in Australia.

**Discussion:**

Significant advances and innovative approaches are needed to address the challenges of accessing healthy food in rural areas.

**Conclusions:**

The resulting WBP has the potential to work at scale for communities in Australia and internationally in designing effective place‐based solutions.

**Summary**

- What is already known on this project
  ○ Increased good access to healthier food retail will be critical to public health efforts to reduce diet‐related diseases in rural Australia
  ○ At present rural living Australians have reduced access to healthy food retail, and healthier options are more expensive compared to their urban counterparts.
  ○ Successful, efforts to make food retail environments healthier need to be true to the context which requires strong commitment to community‐based participation in the co‐design of any intervention or activity.
  ○ New ways are needed to engage a diverse and representative sample of participants from rural communities in participatory research.
- What this study adds to our knowledge
  ○ A Human Centred Design approach can guide the design of a framework to co‐design, test and implement a web‐based platform in rural Australian communities that aims to understand the unique factors influencing access to healthy and unhealthy food, and generate potential solutions for supporting healthier diets.

## 1 Introduction

Providing good access to healthier food retail is critical if diet‐related diseases are to be reduced in rural Australia [1]. A push for greater research investment into improving access to healthy food in rural Australia has been made [2, 3]. The five dimensions of food access include availability (e.g., adequacy of supply or presence of or number of food retailers near home), accessibility (e.g., ability to travel to access food), affordability (e.g., food price), acceptability (e.g., foods meet personal standards and preferences) and accommodation (e.g., appropriate opening hours for community needs) [4]. Rural living Australians have reduced access to healthy food retail compared to their urban counterparts [5, 6, 7, 8]. Healthier options are more expensive for rural populations [5, 7, 9, 10]. In Australia, research highlights disparities in access to healthy foods, albeit with the vast majority focusing on urban areas [6, 11, 12]. Limited research in rural contexts exposes disparities in access to healthy food, with even fewer studies exploring interventions or strategies to improve access in these areas [3, 13].

Internationally, several initiatives are emerging to help overcome challenges to poor access to healthy food in rural communities by increasing availability and accessibility [14, 15, 16, 17, 18]. Research efforts are deliberately trying to address the unique geographic challenges (e.g., transport distances), lifestyle and socioeconomic differences (e.g., access to public transport, employment and education opportunities and access to health services) faced by rural communities to improve health outcomes [16, 19, 20, 21]. Many of these studies emphasise collaboration between the existing human capital in communities (e.g., community members, rural nutrition and dietetics professionals and public health researchers) to generate evidence regarding issues faced by rural communities [2, 18]. This commitment to community‐based participation in co‐design is predicated on the realisation that, to be successful, efforts to make food retail environments healthier need to be true to the context and place [18].

The roll‐out of national broadband across Australia and new technologies such as low‐orbit satellites have provided rural communities with high‐quality internet access for the first time. Increased digital access means Web‐Based Platforms (WBP) (i.e., software applications or services available through a web browser over the internet) can now be used to engage rural populations in research activities, including the co‐design of public health interventions, and represent a critical new avenue for research to improve health in rural communities. Effective engagement through WBPs must ensure employed techniques are sensitive to the unique issues affecting rural communities [18]. By co‐designing a WBP and using participatory techniques [22] we can build and adapt existing evidence‐based WBPs [23, 24, 25] for use in rural communities to identify local solutions to important health issues.

Participatory techniques such as Participatory Mapping (PM) [26] and Group Model Building (GMB) [27] have been used successfully to understand the complex interplay of factors that influence non‐communicable diseases (e.g., healthy eating, active living and purchasing behaviours) and identify potential solutions [28, 29, 30]. As a method, PM provides a space for participants to identify and define the issues, ideas and experiences that are important to them through representation on a map of the geographical areas of interest, which can lead to new understandings of an issue in a specific locality, including the influences of wider social, political, and economic forces on the determinants of health [22]. Similarly, through a series of three workshops, the GMB process develops a systems map of the factors (i.e., a causal loop diagram) that influence a complex problem and how they interact, which can be used to guide the generation and prioritisation of action ideas to improve the complex issue at hand [27]. Combined, PM and GMB have the potential to provide a shared understanding of complex problems and potential solutions being faced specific to the context within one geographical location.

Successfully running PM and GMB research in communities requires careful planning and participant recruitment [31, 32]. Further challenges include engaging with the community, gaining commitment to the time required to participate in workshops, and facilitation skills to manage the dynamics of the workshop [22, 31, 32]. While moving online to run GMB workshops became more prevalent during the Covid‐19 pandemic, the challenges surrounding attendance still apply, with participants required to be available at the same time and have access to a stable internet connection [26]. Reviews have called for new ways to engage diverse participants, including vulnerable populations, to ensure all participants have an equal chance to contribute (i.e., through the use of strategies to address and manage the power imbalance between participants and the research team) [26].

In 2024, a collaboration of public health nutrition researchers, health services, primary health networks and local governments across the Australian states of New South Wales and Victoria set out to co‐design a web‐based platform (WBP) to engage communities using PM and GMB. This WBP aims to understand the factors that influence healthy food access, including geographical access, and provides the opportunity to identify potential solutions. This proposed WBP will be accessible online via a computer, tablet, or phone and will be ‘participant‐paced’, allowing community members to engage with the platform at their own home or in a convenient location (e.g., community library). It is hoped the features of the WBP will enable an increased number of community members to engage with researchers by removing barriers to participation (e.g., time, location and access to transport).

This paper outlines the co‐design framework that will be used to design, prototype, test and implement a WBP that applies PM and GMB approaches to engage with rural communities.

## 2 Methods and Process

### 2.1 Design

This is a Participatory Action Research project that will follow best practice guidelines [33].

Inspired by our project partners and the potential for using PM and GMB in new and innovative ways, we will use a human‐centred design (HCD) (Box 1) approach to ideate, prototype, test and implement a WBP aimed at engaging with rural communities. The objective of the WBP is to understand the unique factors influencing access to healthy and unhealthy food, and potential solutions for supporting healthier diets [34, 35]. The use of HCD was selected as it has been successfully used in earlier research as a problem‐solving approach to developing, refining and testing digital products, programs and services to improve population health [34, 36].

BOX 1 **Human‐centred design: An opportunity to design digital tools to engage rural communities to improve access to healthy food in rural Australia.**

- Rural and remote Australians have reduced access to healthy food retail compared to their urban counterparts, with healthier options often more expensive, affecting both food insecurity and the health of rural residents.
- Calls for greater research investment into improving access to healthy food in rural Australia have been made.
- Studies emphasise that collaboration between the existing human capital in communities (e.g., community members, rural nutrition and dietetics professionals and public health researchers) is needed to better understand issues faced by rural communities.
- Future research needs to increase engagement of diverse participants, including vulnerable populations, ensuring all participants contribute to public health solutions.
- Combined, Participatory Mapping and Group Model Building methods have the potential to provide a shared understanding of complex problems and potential solutions being faced specific to the context of one geographical location.
- The use of web‐based platforms to engage with rural communities has the potential to overcome some of the context specific challenges to participating in research for rural communities.

### 2.2 Setting

In Australia, the Modified Monash Model characterises a location as metropolitan, rural, remote or very remote [37]. This measure of rurality has been used in earlier studies exploring children's weight and dietary health, and has a scale range from Modified Monash (MM)1 (Metropolitan) to MM 7 (Very Remote) [38]. Rural areas are classified as those communities from MM 3 areas and above (MM 3–MM 5). This project will be set in two local government areas in Australia: (1) Colac Otway Shire located in West Victoria, which comprises two different levels of remoteness: one ‘Medium Rural Town’ (MM 4) and several ‘Small Rural Towns’ (MM 5) [5, 37] and (2) Tamworth Regional Council in NSW, comprising two different levels of remoteness: one ‘Large Rural Town’ (MM 3) and multiple ‘Small Rural Towns' (MM 5)’, with a focus on MM 5 residents [37]. These two towns were selected due to key stakeholders demonstrated commitment to improving public health nutrition.

### 2.3 Project Participants

This project is supported by a Medical Research Future Fund Incubator Grant (ID: 2030589) and the Research Team including Chief Investigators and Associated Investigators with representation from Colac Otway Shire in Victoria and Tamworth Regional Council in New South Wales. Participants in the co‐design of the WBP will include the research team, as well as key stakeholders and community members located in the two local government areas (LGAs). These participants will form the Project Steering and Community Advisory Groups (PSG and CAG), who will be responsible for co‐leading all project phases.

#### 2.3.1 PSG

The PSG, established upon project commencement, will provide support, guidance and oversee the progress of the project. The PSG will comprise the Research Team, including Chief Investigators and Associate Investigators, which collaborated on the successful grant application that funded this work (*n* = 19). The Research Team includes experts in the field of systems science, co‐design, public health nutrition, geospatial science and software engineering, as well as representatives from LGA councils, health services and primary health networks. In addition, place‐based researchers who live and/or work closely with these communities will be employed and play a role in leading the PSG.

#### 2.3.2 CAG

The CAG will include community representatives from Colac and Tamworth LGAs who will work closely with the Research Team to co‐design and test the WBP and assist with the development of a tailored community engagement plan for each LGA. The PSG and place‐based researchers will identify key community members to invite to the CAG to provide area‐level insight into the project. It is expected that this will include 10–15 people who live and/or work in each LGA within the community. It is expected that the CAG will be composed of people who are linked to food relief agencies, community and neighbourhood houses, libraries and schools. Appropriate remuneration for the time of the CAG will be allocated as they participate in each phase.

### 2.4 Procedure

**FIGURE 1:**
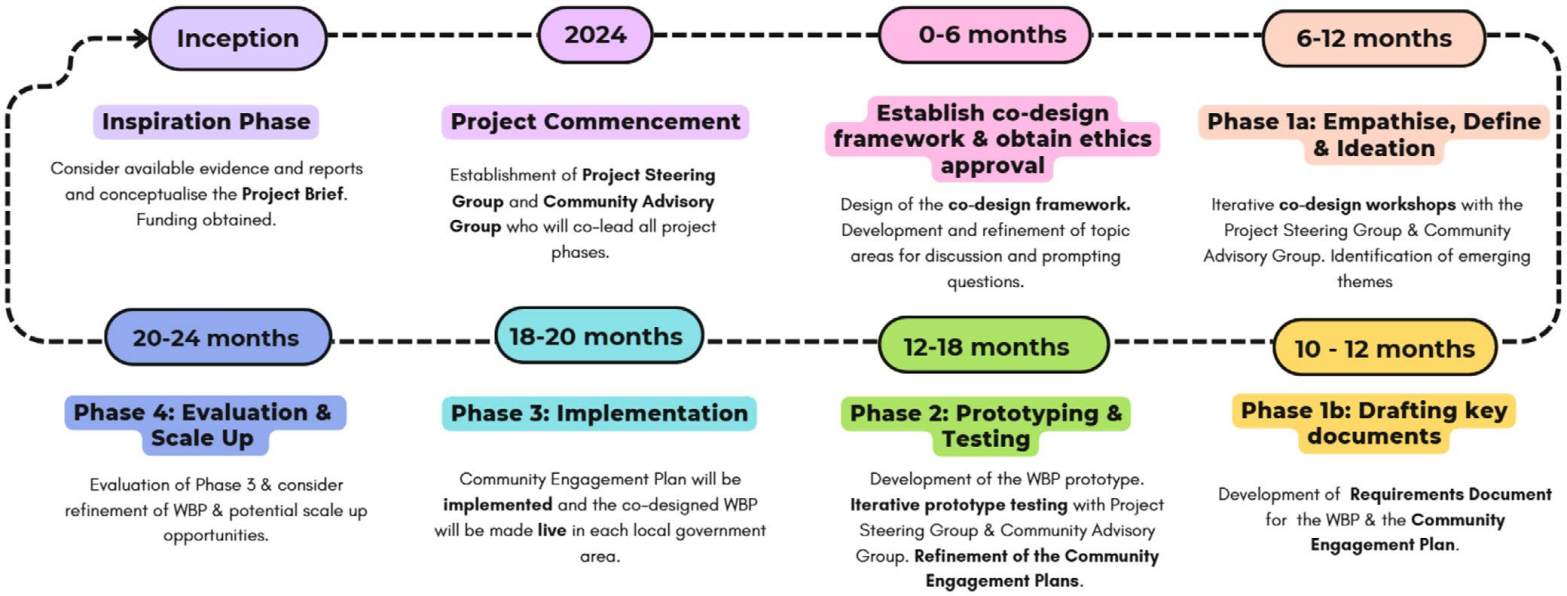
Project timeline for the human‐centred design of a web‐based platform to engage rural communities.

This project will take a HCD approach [34, 36] structured into four phases described below (Figure 1). It should also be noted that prior to the commencement of this project, there was an ‘Inspiration’ phase (not discussed in this paper) during which the Research Team considered the available evidence and reports and conceptualised the successful grant application that funded this project.

#### 2.4.1 Phase 1—Empathise, Define and Ideation

This phase of the co‐design process incorporates the HCD thinking model steps of ‘Empathise’ (i.e., understanding who our users are by learning about them and their needs), ‘Define’ (i.e., identifying what is needed to make the WBP successful), and ‘Ideation’ (i.e., generating ideas to build a successful WBP) [35]. These phases will directly inform the design features and functionality of the WBP and the development of a community engagement strategy for each LGA [36]. This phase will involve one to two co‐design workshops (2–3 h each) with the CAG at each research site (i.e., up to two workshops in each LGA) as well as two PSG workshops, which will be delivered via hybrid meetings (i.e., mix of online and in person attendees). Participants will receive a project brief (i.e., summary of the project grant proposal, PM and GMB methodologies) with an invitation to attend the co‐design workshop. In the co‐design workshop itself, participants will be provided with a short overview of the project brief and then take part in a facilitated discussion on six key areas: (1) Who are our users and what do we want to know; (2) What is the big question; (3) Usability and feedback mechanisms; (4) Participatory mapping indicators; (5) Community engagement; and (6) Impact and evaluation. Each key area will be introduced to participants, followed by prompting questions for discussion. Table 1 presents the questions which were developed and refined by the Research Team.

**TABLE 1:** Topic areas and promoting questions for a co‐design workshop developing a web‐based platform using a human‐centred design approach.

Topic	Human‐centred design phase [26]	Prompting questions
Q1: Who will be our users & what do we want to know?	Empathise: Understand who the users of the web‐based platform will be, their needs, challenges and experiences	Who are the primary users of the digital tool (e.g., community members, planners, researchers)? What do we want to know about them for this to be useful? E.g., gender, employment, age, parent? How can the tool facilitate collaboration and engagement among users?
Q2: What is the big question?	Define: Understand the key challenge and opportunity that we want to address	What specific goals or outcomes are we aiming to achieve through use of the digital tool? What are the key factors we want to understand? What is it that we could measure or would measure if we were winning?
Q3: Usability and feedback mechanisms	Ideation: Generation of ideas that can inform the design and functionality of the Web‐Based Platform	What accessibility features should be considered to ensure inclusivity (e.g., multi‐language support, adaptive interfaces)? What design principles should guide the design of the tool to ensure usability and accessibility? Are there opportunities to customization or personalization the digital tool to enhance experience and engagement?
Q4: Participatory Mapping—indicators	Define: Understanding the important geographical information that we want to capture from participants to understand the barriers and facilitators to accessing healthy and unhealthy food in their local government area	What are the ‘spatial indicators and/or information’ that you would like to draw from the participatory mapping process to gauge the communities lived experience of the rural food retail environment. What are the important places on the map that relate to food we need to know about (e.g., supermarkets and fast‐food)? What are the important places on the map people travel from to access food we need to know about (e.g., home and work)?
Q5: Community engagement	Ideation: Generation of ideas for how best to engage the local community in each area to use to the web‐based platform when implemented	How can the tool encourage participation and input from diverse stakeholders? What are our community engagement strategies/communication strategies for our two towns? What mechanisms should be in place to ensure the accuracy and reliability of user‐contributed data? Are there opportunities to integrate feedback loops or gamification elements to incentivise participation?
Q6: Impact and evaluation	Define: Understanding how the impact of the web‐based platform can be measured and evaluated	How will the effectiveness of the participatory mapping tool be evaluated? What metrics or indicators will be used to measure the tool's impact on decision‐making processes or community outcomes? Are there mechanisms for continuous improvement based on user feedback and evolving needs?

Workshops will be recorded and deductively thematically analysed using NVivo (Version 14) to understand and define key outputs of each step, which will inform Phase 2. Each workshop preceding the first co‐design workshop will involve consensus checking and refinement of emerging themes from co‐design workshops. When consensus is reached at the end of Phase 1, a ‘Requirements Document’ (RD) will be drafted outlining the requirements of the WBP with a step‐by‐step guide of what it is envisaged the user will see as they progress through using the WBP. In this phase, a Community Engagement Plan will be developed to guide the implementation phase for each LGA.

#### 2.4.2 Phase 2—Prototyping and Testing

In Phase 2, the RD will be used by the software engineer to build the WBP prototype. In this phase, the Community Engagement Plan will also be presented to the CAG and PSG for consideration and refinement. Once the WBP prototype is developed, it is expected that there will be two to three rounds of prototype testing with the CAG and the PSG. Prototype testing with the PSG will take place at regular 90‐min quarterly meetings and will involve the following: (1) WBP development progress update, (2) demonstration and/or testing of the WBP, (3) a discussion around the WBP functionality of the WBP, and (4) a review of the Community Engagement Plan. Feedback will be captured by notetakers and incorporated into the next iteration of the RD for further development of the WBP prototype. Prototype testing with the CAG will take place in the community, and additional community members will be engaged where possible through CAG networks. The CAG testing will provide an opportunity for participants to use the WBP as if they were participants in the ‘Implementation Phase’ and provide feedback on the prototype via an online survey at the completion of the WBP and in discussion with the Research Team. Feedback from the CAG will be captured by notetakers and incorporated into the next iteration of the RD for further development of the WBP prototype.

#### 2.4.3 Phase 3—Implementation

In this phase, the Community Engagement Plan will be implemented, and the co‐designed WBP will be made live in each LGA. It is expected that communities may be engaged through various methods such as social media campaigns, engagement with local schools (primary and secondary) through classroom‐based activities, and through engagement with Public Health Units/Health Service Networks and local government staff. In addition, QR codes will be placed in community locations to access the WBP. The implementation/pilot phase will run over a 2‐ to 3‐month period with an aim to reach 1000 people per LGA (*n* = 2000 total).

#### 2.4.4 Phase 4—Evaluation and Scale‐Up

The Research Team will evaluate Phase 3 using the RE‐AIM (Reach Effectiveness Adoption Implementation and Maintenance) framework [39] to establish the success of the WBP. Measures will include, at a minimum: the number of community members that used the WBP tool and the cohort they represent, geographic spread (reach), and assessment of whether participants completed the full spectrum of activities through the WBP (adoption), and whether the findings inform policy or program changes or secure funding to support implementation (maintenance). We will also explore the differences in participation across LGAs to understand the sustainability and scalability of the WBP for use in other communities and countries.

Low‐risk ethics approval for Phases 1 and 2 has been granted for this project by the lead authors' institution (HEAG‐H 111_2024) and partner organisation's institution (R‐2024‐0053).

## 3 Discussion

Significant advances and innovative approaches are needed to address the challenges of accessing healthy food in rural areas. These advances are critical if we are to address the inequitable burden of diet‐related diseases experienced by rural communities. Taking a HCD approach, this project outlines a framework for co‐designing an interactive WBP, which seeks to engage a diverse range of people across two rural communities in Australia to participate in research on improving healthy food access (Box 2). The resultant world‐first tool (the WBP) will provide a platform for communities to generate local solutions and drive change for diverse populations within rural communities in Australia and internationally.

BOX 2 **Co‐designing digital participatory approaches to improve health in rural Australia.**

This paper presents an innovative human‐centred approach to co‐designing, prototyping and testing a web‐based platform (WBP) and community engagement plan. This approach seeks to enable broad community engagement in rural research by using digital solutions to remove participation barriers such as time, travel and logistics, as well as the human resources requirement to deliver participatory research in person. We present a co‐design framework that will be delivered in two rural local government areas in Australia to develop an interactive WBP. This WBP aims to support the creation of healthier food retail opportunities in rural and remote Australia. The resulting WBP will have the potential to work at scale to enable communities to actively be involved in identifying local barriers and facilitators to important public health issues and generating effective place‐based solutions.

## Author Contributions

**Cindy Needham:** conceptualization, methodology, investigation, project administration, visualization, funding acquisition, writing – original draft. **Jane Jacobs:** conceptualization, methodology, funding acquisition, writing – review and editing. **Christina Zorbas:** conceptualization, methodology, funding acquisition, writing – review and editing. **Laura Alston:** conceptualization, methodology, funding acquisition, writing – review and editing. **Tracy Schumacher:** conceptualization, methodology, funding acquisition, writing – review and editing. **Penny Fraser:** conceptualization, methodology, funding acquisition, writing – review and editing. **Ana Horta:** conceptualization, methodology, funding acquisition, writing – review and editing. **Michael Johnstone:** conceptualization, methodology, funding acquisition, writing – review and editing. **Douglas Creighton:** conceptualization, methodology, funding acquisition, writing – review and editing. **Alison Koschel:** conceptualization, methodology, funding acquisition, writing – review and editing. **Leanne J. Brown:** conceptualization, methodology, funding acquisition, writing – review and editing. **Annabelle Williams:** conceptualization, methodology, funding acquisition, writing – review and editing. **Judy Coates:** conceptualization, methodology, funding acquisition, writing – review and editing. **Gina Vereker:** conceptualization, methodology, funding acquisition, writing – review and editing. **Carmen Vargas:** conceptualization, methodology, funding acquisition, writing – review and editing. **Claudia Strugnell:** conceptualization, methodology, funding acquisition, writing – review and editing. **Tari Forrester‐Bowling:** conceptualization, methodology, funding acquisition, writing – review and editing. **Kristy A Bolton:** conceptualization, methodology, funding acquisition, writing – review and editing. **Steven Allender:** conceptualization, methodology, funding acquisition, writing – review and editing.

## Ethics Statement

Low‐risk ethics approval has been granted for this project by the Deakin University Human Ethics committee (HEAG‐H 111_2024) and the University of Newcastle Human Ethics committee (R‐2024‐0053).

## Conflicts of Interest

The authors declare no conflicts of interest.

## Data Availability

The authors have nothing to report.
